# Genome-Wide Identification of miRNAs Responsive to Drought in Peach (*Prunus persica*) by High-Throughput Deep Sequencing

**DOI:** 10.1371/journal.pone.0050298

**Published:** 2012-12-05

**Authors:** Vahap Eldem, Ufuk Çelikkol Akçay, Esma Ozhuner, Yakup Bakır, Serkan Uranbey, Turgay Unver

**Affiliations:** 1 Cankırı Karatekin University, Faculty of Science, Department of Biology, Cankiri, Turkey; 2 Istanbul University, Faculty of Science, Department of Biology, Istanbul, Turkey; 3 Suleyman Demirel University, Faculty of Agriculture, Department of Agricultural Biotechnology, Isparta, Turkey; 4 Marmara University, Faculty of Arts and Science, Department of Biology, Istanbul, Turkey; Virginia Tech, United States of America

## Abstract

Peach (*Prunus persica* L.) is one of the most important worldwide fresh fruits. Since fruit growth largely depends on adequate water supply, drought stress is considered as the most important abiotic stress limiting fleshy fruit production and quality in peach. Plant responses to drought stress are regulated both at transcriptional and post-transcriptional level. As post-transcriptional gene regulators, miRNAs (miRNAs) are small (19–25 nucleotides in length), endogenous, non-coding RNAs. Recent studies indicate that miRNAs are involved in plant responses to drought. Therefore, Illumina deep sequencing technology was used for genome-wide identification of miRNAs and their expression profile in response to drought in peach. In this study, four sRNA libraries were constructed from leaf control (LC), leaf stress (LS), root control (RC) and root stress (RS) samples. We identified a total of 531, 471, 535 and 487 known mature miRNAs in LC, LS, RC and RS libraries, respectively. The expression level of 262 (104 up-regulated, 158 down-regulated) of the 453 miRNAs changed significantly in leaf tissue, whereas 368 (221 up-regulated, 147 down-regulated) of the 465 miRNAs had expression levels that changed significantly in root tissue upon drought stress. Additionally, a total of 197, 221, 238 and 265 novel miRNA precursor candidates were identified from LC, LS, RC and RS libraries, respectively. Target transcripts (137 for LC, 133 for LS, 148 for RC and 153 for RS) generated significant Gene Ontology (GO) terms related to DNA binding and catalytic activites. Genome-wide miRNA expression analysis of peach by deep sequencing approach helped to expand our understanding of miRNA function in response to drought stress in peach and Rosaceae. A set of differentially expressed miRNAs could pave the way for developing new strategies to alleviate the adverse effects of drought stress on plant growth and development.

## Introduction

Peach (*Prunus persica* L.) is considered to be one of the most widely grown and economically important stone fruit species in the Rosaceae, comprising more than 3,000 species in approximately 110 genera distributed worldwide [1]. In 2010, it was estaminated that world annual production of peaches and nectarines exceeded 19 million metric tons according to FAO statistics (FAOSTAT, http://faostat.fao.org). In addition to its ecological and high economic importance, peach is also emerging as a model tree species for both comparative genomic studies, evolutionary studies and plant development research owing to its small genome size of 300 Mb (just about twice comparing with *Arabidopsis thaliana*) and the relatively short reproductive time [2], [3]. Peach has a haploid chromosome number of 8 [4] and the first draft of peach genome (peach v1.0, obtained from “Lovell” haploid) was unraveled by the International Peach Genome Initiative (IPGI), which are available from the Genome Database for Rosaceae (http://www.rosaceae.org/peach/genome). The genus *Prunus*, which includes peach, nectarine, apricot, weet and sour cherry, have stone fruits with fleshy mesocarp, but the growth and development of these fruits, especially large-fruited species like peach, are seriously affected by drought. For peach, drought stress is one of the major abiotic stresses limiting fruit production and quality during the 4–6 week period before harvesting [5], [6].

As a major abiotic factor, drought can be described as basically the water deficiency or insufficient access to water and it has adverse effects on the growth of plants and crop production. However, plants growing in drought stress respond to dehydration and have to develop a variety of mechanisms at morphological and molecular level in order to remain alive. Deciphering the physiological processes and molecular genetic mechanisms involved in drought resistance has certainly made a significant progress in understanding of complex biological response of plants at the molecular and organism levels against the drought. The expression profile of protein-coding genes could highly fluctuate in response to drought at the transcriptional and post-transcriptional levels of miRNA [7], [8], [9]. The aberrant expresison of genes may be regulated by an newly discovered small RNAs, termed microRNAs (miRNAs).

The miRNAs are an extensive class of small (19–25 nucleotides), single-strand, endogenous, noncoding RNAs which negatively modulate gene expression at the post-transcriptional levels by directing the cleavage of mRNAs or by inhibiting translation depending on the extent of complementarity between miRNA and its target(s) [7], [8], [10]–[13]. In plants, biogenesis of miRNA necessaties a multiple biological process to generate full-function mature miRNAs by recruiting several evolutionary conserved protein families. At first, plant miRNA genes are transcribed to primary miRNAs (pri-miRNAs) by RNA polymerase II [14]–[16], then these long pri-miRNAs are cleaved to hairpin-like miRNA precursor (pre-miRNAs) and the loop-regions of the hairpin are excised by RNAse III enzyme DICER LIKE1 (DCL1) [17] with the aid of HYL1 and SERRATE [18]–[21]. Eventually, released mature miRNAs incorporate into ribonucleoprotein complex known as the RNA-induced silencing complex (RISC), which inhibits translation elongation or triggers the degradation of target mRNA [12], [22].

Considerable amount of plant miRNAs have been identified by computational and/or experimental methods and these miRNAs have been deposited in the latest release of miRBase v18 (release 18.0 November 2011, only experimentally validated [23]) and PMRD (Plant microRNA database, both experimental and computational [24]) since the first miRNAs were discovered in plants in 2002 [25], [26]. At present, there are 4053 hairpin entries pertaining to 52 plant species in the miRBase v18.0. The identification of any miRNAs has great importance for subsequent research such as miRNA function, nature, target prediction and biogenesis. In recent years, the innovative strategies and practical methodologies have been developed for determining miRNA expression. The main approaches of experimental methodologies can be summarized as follows: direct-cloning [27]–[29], stem-loop qRT-PCR [10], [30]–[32], next-generation sequencing technology [33]–[37] and hybridization-based detection, such as northern blotting [38], *in situ* detection [39], [40] and microarray [31], [41], [42]. Although each of these methods has their own particular advantages and disadvantages, the next-generation sequencing technologies play an increasingly prominent role in discovering novel miRNAs [36], [43] and measuring quantitively expression levels of low-abundant [44] and species or tissue specific miRNAs [35], [45] comparing to other genome-wide transcriptome analysis methods, such as miRNA-microarray [46], [47]. In addition to experimental approach, computational approach has also been a preferred method because of its low cost, high efficiency and speed, prior to experimental validation. The efficacy and power of computational approach come from its major characteristic features: (i) high evolutionary conservation from mossess to eudicots in plants (comparative genomics) [48], [49], (ii) hairpin-shaped stem-loop secondary structure [50], [51], (iii) high minimal folding free energy index [48].

Many recent studies have revealed that plant miRNAs have pivotal roles in plant response to abiotic stresses, including drought [9], [52], [53], salt [54], [55], [56], cold [33], [57], oxidative stress [58]–[60] and UV-B radiation [61]. The miRNAs whose expression level is significantly altered in drought condition compared with normal conditions have been well-reviewed in recent works [7], [8]. It has been indicated that a certain number of miRNAs involve in response to drought stress by altering the gene expression [52], [62], [63]. As for *Prunus* species, there is no comprehensive data about the expression profiles of drought responsive miRNAs. The aim of the present study is to determine the expression profile of drougt stress-responsive miRNAs in peach. Thus miRNA deep sequencing by Illumina HiSeq 2000 were applied not only for simultaneous evaluation of drought responsive miRNAs' expressions, but also for providing comprehensive information about *P. persica* miRNA transcriptome on genome-wide scale. Stem-loop real time qRT-PCR (ST-RT PCR) was also employed to futher validate the expression level of a set of miRNAs identified during deep sequencing. Additionally, the identification and characterization of *P. persica* miRNAs and their target genes were established by using computational methods combined with experimental validation.

## Results

**Figure 1:**
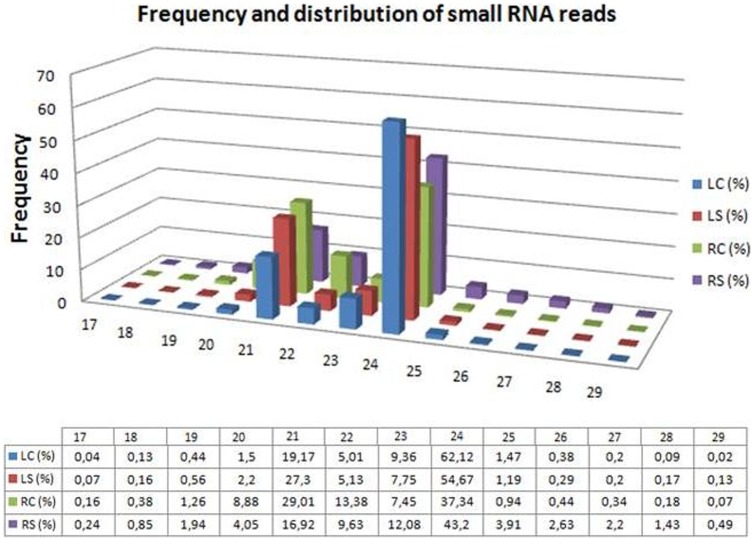
The length distribution of small RNA sequences from each library of leaves and roots after pre-drought and post-drought treatment. The y axis indicates the frequency of number of small RNA reads in each library whereas the x axis corresponds to the nucleotide (nt) numbers of small RNA length.

We used the Illumina Solexa sequencing platform to investigate the genome-wide identification and expression profiles of miRNAs in peach, particularly for the drought-responsive miRNAs. Four small RNA libraries were constructed by the use of total RNAs isolated from control leaf (LC), drought-stressed leaf (LS), control root (RC), and drought-stressed root (RS) tissues. Small-RNA sequencing yielded a total of 53,878,885 high-quality raw sequence. Total high-quality raw reads in each of LC, LS, RC and RS libraries are 15,499,314, 12,473,137, 12,703,130 and 13,203,304, respectively (Table 1). After removing low quality reads, adapters, poly-A sequences and short RNA reads smaller than 18 nucleotides, 53,475,533 (99.23%) clean reads including 14,204,383 unique sequences were obtained from the all libraries. Among the unique sequences, 2,063,684 (49.01%), 1,599,019 (50.40%), 1,400,836 (51.96%) and 1,747,201 (42.36%) were mapped to the peach genome using SOAP2 for sequences generated from LC, LS, RC and RS, respectively (Table 1 and Table S1). In order to get a big-picture view of sequence distribution of all sRNA reads, all clean reads were mapped against the peach genome database at Genbank (http://www.ncbi.nlm.nih.gov/genome/388), Rfam (http://rfam.sanger.ac.uk/) and miRBase v18.0 (http://www.mirbase.org/), and therefore, they are classified into seven annotation categories: non-coding RNAs (tRNA, rRNA, snRNA and snoRNA), miRNA, exon-sense, exon-antisense, intron-sense, intron-antisense, and unknown sRNAs (Table 2, Figure S1). As shown in Table 2, the highest abundance of unique conserved and potential non-conserved miRNAs reads was found in root-drought stress library and leaf control library, whereas most of the total miRNA reads were found in leaf-stress library. The length distribution of unique sRNA reads revealed that the majority of reads from each library were 18–25 nt in length, of which the class of 24 nt was the most abundant group accounted for average ∼50% of total reads for each library and it was followed by the group of 21 nt class (Figure 1). Although these small RNAs unevenly distributed in four groups according to their length, small RNAs in control and drought-exposed group for leaf and root represent similar distribution within each of their own group (Figure 1).

**Table 1:** Statistics of small RNA sequences for control and drought stress libraries from *Prunus persica* leaf and root.

Library	Raw reads	High-quality reads	Clean reads	Unique sRNAs	Total sRNAs mapped to Genome	Unique sRNAs mapped to Genome
LC	15,521,503	15,499,314	15,470,689	4,210,911	10,264,244 (66.35%)	2,063,684 (49.01%)
LS	12,492,645	12,473,137	12,428,654	3,172,346	8,673,228 (69.78%)	1,599,019 (50.40%)
RC	12,726,680	12,703,130	12,539,747	2,696,057	9,324,699 (74.36%)	1,400,836 (51.96%)
RS	13,233,471	13,203,304	13,036,443	4,125,069	8,157,867 (62.58%)	1,747,201 (42.36%)

**Table 2:** Classification of small RNA sequences from control and drought stress libraries.

	LC		LS		RC		RS	
Category	Unique (%)	Total (%)	Unique (%)	Total (%)	Unique (%)	Total (%)	Unique (%)	Total (%)
Exon antisense	66583 (1,58)	233813 (1,51)	53440 (1,68)	176740 (1,42)	68331 (2,53)	162078 (1,29)	61648 (1,49)	168170 (1,29)
Exon sense	104977 (2,49)	534317 (3,45)	85103 (2,68)	403706 (3,24)	102294 (3,79)	321626 (2,56)	115637 (2,80)	379058 (2,90)
Intron antisense	73179 (1,73)	336082 (2,17)	54652 (1,72)	234564 (1,88)	48174 (1,78)	170265 (1,35)	60327 (1,46)	219032 (1,68)
Intron sense	99820 (2,37)	736794 (4,76)	76356 (2,40)	508059 (4,08)	68976 (2,55)	384332 (3,06)	82342 (1,99)	419902 (3,22)
miRNA	28239 (0,67)	2004049 (12,95)	23627 (0,74)	2602064 (20,93)	24359 (0,90)	1971298 (15,72)	31969 (0,77)	963596 (7,39)
rRNA	52275 (1,24)	526784 (3,40)	45662 (1,43)	390134 (3,13)	87516 (3,24)	2223692 (17,73)	202183 (4,90)	2552133 (19,57)
snRNA	2030 (0,04)	7435 (0,04)	1443 (0,04)	4365 (0,03)	4080 (0,15)	34663 (0,27)	3827 (0,09)	24744 (0,19)
snoRNA	732 (0,01)	1619 (0,01)	622 (0,02)	1294 (0,01)	837 (0,03)	3522 (0,02)	1700 (0,04)	6349 (0,04)
tRNA	4841 (0,11)	83704 (0,54)	4822 (0,15)	97317 (0,78)	8763 (0,32)	895226 (7,13)	39282 (0,95)	303823 (2,33)
Unannotated	3778235 (89,72)	11006092 (71,14)	2826619 (89,1)	8010411 (64,4)	2282727 (84,6)	6373045 (50,82)	3526154 (85,48)	7999636 (61,36)

### Identification of known miRNAs in peach

In order to identify known (both conserved and species-specfic) miRNAs from control and drought-exposed root and leaf tissues of peach, small RNA sequences generated from each library were independently aligned with currently known and experimentally validated mature miRNAs deposited in miRBase v18.0, including 4,014 viridiplantae miRNAs belonging to 52 plant species. After homology search, a total of 531, 471, 535 and 487 miRNAs were identified from LC, LS, RC and RS libraries, respectively (Table S2). These miRNAs belong to 43 evolutionary conserved miRNA families (Table 3), suggesting that miRNA-mediated biological process are also present in peach as found in other plant species. However, some miRNAs, such as miR416, miR437, miR441 and miR529, were not detected in both leaf and root samples, suggesting that these miRNAs may be tissue-specific expression. The expression levels varied from miRNAs to miRNAs from one copy to more than one million of copies based on the deep sequencing (Table S2). A majority of miRNAs were detected with more than 50 copies; such as a total of 272 miRNAs for LC (51,22%), 229 miRNAs for LS (48,61%), 225 miRNAs for RC (46,20%) and 269 miRNAs for RS (50,28%) were sequenced more than 50 times. As previously reported, evolutionary conserved miRNAs have generally high expression abundances when compared with non-conserved miRNAs. Among the conserved miRNAs, total reads of miR535, miR157, miR166, miR156 and miR408 accounted for vast majority of total miRNAs; LC (82,49%), LS (89,09%), RC (75,54%) and RS (55,02%). Of these, miR535 was the most abundant miRNA in both control and drought-exposed libraries (Table S2).

**Table 3:** List of conserved miRNAs obtained from control and drougt-stresses leaves and roots of *P. persica*.

miRNA Family	miRNA sequence (5′-3′)	Length (nt)	Count				Fold Change (Log2)		miRNA Orthologs					
			LC	LS	RC	RS	LC vs LS	RC vs RS	*Ath*	*Osa*	*Ptc*	*Mtr*	*Rco*	*Vvi*
miR156	UGACAGAAGAGAGUGAGCAC	20	934112	1346985	68360	81033	0.84	0.18	+	+	+	+	+	+
miR157	UUGACAGAAGAUAGAGAGCAC	21	262981	465762	606233	87444	114.05*	−284.94*	+	+	+	+	−	−
miR159	UUUGGAUUGAAGGGAGCUCUA	21	3033	1889	2176	489	−0.36	−220.98*	+	+	+	+	+	+
miR160	GCGUACGAGGAGCCAAGCAUA	21	2969	2235	2118	13332	−0.09	259.80*	+	+	+	+	+	+
miR162	UCGAUAAACCUCUGCAUCCAG	21	321	310	261	92	0.26	−156.03*	+	+	+	+	+	+
miR164	UGGAGAAGCAGGGCACGUGCA	21	13187	13085	9571	3088	0.304	−168.80*	+	+	+	+	+	+
miR166	UCGGACCAGGCUUCAUUCCCC	21	144793	176391	158327	109345	0.60	−0.59	+	+	+	+	+	+
miR167	UGAAGCUGCCAGCAUGAUCUG	21	92812	71576	83715	5571	0.058	−369.55*	+	+	+	+	+	+
miR168	UCGCUUGGUGCAGGUCGGGAA	21	20085	21168	14751	16549	0.391	0.109	+	+	+	+	+	+
miR169	UGAGCCAAGAAUGACUUGCUG	21	907	804	1420	94	0.141	−397.31*	+	+	+	+	+	+
miR171	UUGAGCCGCGUCAAUAUCUCC	21	262	128	90	393	−0.707	207.08*	+	+	+	+	+	+
miR172	AGAAUCUUGAUGAUGCUGCAU	21	2874	1891	1719	1212	−0.288	−0.560	+	+	+	+	+	+
miR319	UUGGACUGAAGGGAGCUCCC	20	106	124	83	9	0.542	−326.110	+	+	+	+	+	+
miR390	AAGCUCAGGAGGGAUAGCGCC	21	4098	5471	1862	2659	0.732	0.457	+	+	+	+	+	+
miR395	CUGAAGUGUUUGGGGGAACUC	21	120	42	Not Detected	−119.869*	−	+	+	+	+	+	+	
miR396	GCUCAAGAAAGCUGUGGGAGA	21	2993	3033	3388	539	0.355	−270.811*	+	+	+	+	+	+
miR397	UCAUUGAGUGCAGCGUUGAUG	21	10464	11428	36291	4151	0.443	−318.412*	+	+	+	−	+	+
miR398	UGUGUUCUCAGGUCGCCCCUG	21	1741	1569	10259	586	0.165	−418.588*	+	+	+	+	+	+
miR399	UGCCAAAGAAGAGUUGCCCUA	21	103	91	36	27	0.137	−0.471	+	+	+	+	+	+
miR403	UUAGAUUCACGCACAAACUCG	21	140	144	109	54	0.356	−106.935*	+	−	+	−	+	+
miR408	ACAGGGAACAGGUAGAGCAUG	21	77621	55268	71947	34715	−0.174	−110.741*	+	+	+	+	+	+
miR414	GCAUCCUCAUCAUCAUCGU	19	Not Detected	58	10	−	−259.206*	+	+	+	−	−	−	
miR415	AAAGAUCCAGAAACAGAGCAG	21	806	516	2	24	−0.327	352.886*	+	+	−	−	−	−
miR418	UAUGUUGAUGAUGAAGAGGACG	22	163	138	Not Detected	0.075	−	+	+	−	−	−	−	
miR419	UGAUGAUGCUGACGAUGACGA	21	52	15	54	66	−147.767*	0.233	+	+	+	−	−	−
miR420	AAACUAAACCGGAAACUGCA	20	Not Detected	24	0	−	−758.037*	+	+	−	−	−	−	
miR444	GGUUGUCUCAAGAUUGUCUCC	21	14	149	176	0	372.773*	−1045.485*	−	+	−	−	−	−
miR472	UCUUUCCCAAUCCACCCAUGCC	22	837	676	1408	99	0.007	−388.611*	+	−	+	−	−	−
miR479	UGUGAUAUUGGUUCGGUUCAU	21	43	21	12	428	−0.718	510.039*	−	−	+	−	−	+
miR535	UGACGACGAGAGAGAGCACGC	21	989554	1438413	604358	277182	0.855	−118.061*	−	+	+	−	+	+
miR827	UUAGAUGACCAUCAACAAACA	21	156	51	273	14	−129.711*	−434.146*	+	+	+	−	−	−
miR2118	CUACCGAUUCCACCCAUUCCGA	22	1358	1345	4343	1897	0.301	−125.101*	−	+	−	+	−	−

Abbreviations:LC; Leaf-control, LS; Leaf-stress, RC; Root-control, RS; Root-stress, Ath, *Arabidopsis thaliana*; Osa, *Oryza sativa*; Ptc, *Populus trichocarpa*;

Mtr, *Medicago truncatula*; Rco, *Ricinus communis*; Vvi, *Vitis vinifera*. Note that the asterisk indicates a statistically significant difference between control and drought-stresses samples.

### Identification of novel miRNAs in peach

**Figure 2:**
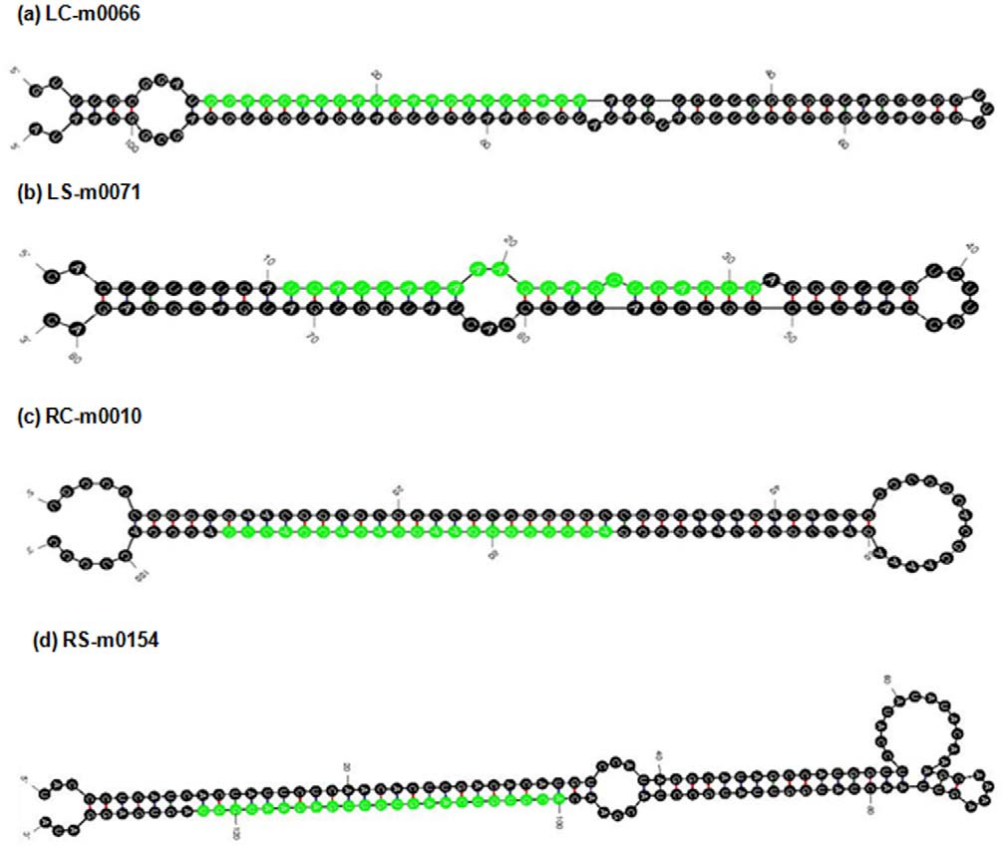
Secondary structure prediction of novel peach miRNA precursors, (a) LC-m0066, (b) LS-m0071, (c) RC-m0030, and (d) RS-m0154.

After obtaining known miRNAs in peach, the remaining sequences of four libraries, which are classified as “unannotated” (excluding known miRNAs and Rfam matching other non-coding RNAs), were taken into consideration to discover novel and potential peach-specific miRNA candidates. To accomplish this, these small RNA sequences were aligned with the *P. persica* genome to identify genomic regions potentially harbouring potential pre-miRNA sequences whose hairpin-like structures are widely used for distinguishing miRNAs from other small non-coding RNAs. The minimum of free energy (MFE) of the secondary structures was also considered to be another criteria for prediction of potential pre-miRNAs. After aligning these unannotated sequences to the genome, we obtained a total of 197, 221, 238 and 265 novel miRNA precursor candidates for LC, LS, RC and RS libraries, respectively (Table S4) and some of these novel miRNA candidates with characteristic features are listed in Table 4. All novel miRNA prediction were carried out according to the default parameters of **MIREAP** (MicroRNA Discovery By Deep Sequencing) software developed by BGI. In agreement with previously reported results, the uracil nucleotide is dominant in the first position of 5′ end for majority of these newly determined putative novel miRNAs. The first nucleotide bias analysis showed that uracil was the most frequently used first nucleotide in miRNAs of *P. persica*; with 10,528 uracil nucleotides (47%) for LC, 12,834 uracil nucleotides (43%) for LS, 21,571 uracil nucleotides (63%) and 13,014 uracil nucleotides (34%) for RS library (Table S3). Our sequence analysis for all libraries showed that the putative pre-miRNAs of each library greatly varied from 70 to 365 nucleoties in length. With the usage of software mFold, these pre-miRNA sequences were applied to predict the characteristic stem-loop secondary structure of pre-miRNA and their locations were also determined in the genomic loci (Tables S4 and S5). Some of the stem-loop secondary structures of predictive pre-miRNAs of *P. persica* determined via mFold can be found in Figure 2. We also calculated the minimum folding free energies of putative peach miRNA precursors for each libraries; ranging from −18,8 to −157,40 kcal/mol with an average of −53,10 kcal/mol for LC, from −18,3 to −171,23 kcal/mol with an average of −55,28 kcal/mol for LS, from −18,32 to −157,4 kcal/mol with an average of −50,99 kcal/mol for RC and from −18,11 to −181,01 kcal/mol with an average of −50,34 kcal/mol for RS (Table S4). In contrast with the common or evolutionarily conserved miRNAs, the predicted novel miRNAs are often expressed at a very low level as reported before. One possible explanation for this result was that many plant miRNAs are evolutionarily conserved and approximately one hundred miRNA conserved miRNA families were found in the Arabidopsis [64]. These evolutionarily conserved miRNAs regulate target transcripts involved in many key metabolic processes which is commonly found in viridiplantea, thus their expression level may be higher than non-conserved miRNAs [65]. Of the 197 putative miRNAs for LC library, only nine miRNAs (LC-m0019, LC-m0049, LC-m0060, LC-m0131, LC-m0146, LC-m0156, LC-m0162, LC-m0164 and LC-m0225) were sequenced more than 500 times, whereas among 221 putative miRNAs belonging to LS library, eleven miRNAs (LS-m0019, LS-m0044, LS-m0051, LS-m0058, LS-m0145, LS-m0181, LS-m0185, LS-m0230, LS-m0234, LS-m0242 and LS-m0256) had more than 500 reads. As for root libraries, eight miRNAs (RC-m0028, RC-m0029, RC-m0077, RC-m0173, RC-m0177, RC-m0200, RC-m0231 and RC-m0253) were sequenced more than 500 reads in control library while eight putative miRNAs (RS-m0025, RS-m0056, RS-m0072, RS-m0153, RS-m0176, RS-m0207, RS-m0244 and RS-m0263) have more than 500 reads in stress library (Table S4).

**Table 4:** Potential novel miRNAs found in *Prunus persica*.

miRNA ID	Sequence (5p)	Sequence (3p)	Length (nt)	Pre-miRNA Length (nt)	Count	Location *	Arm	MFE (kcal/mol)
LC-m0007	-	UUGUUUAAAACCCGUCGUCUCUA	23	186	7	scaffold_1:8956580:8956765	+	−60.70
LC-m0026	GGATTGTTTAGTGTGCGGATT	TACGCATGCTAAACAATCCGG	21	200	248	scaffold_1:2565511:2565710	−	−75.40
LC-m0057	-	CGAAUUAUUUUUCGUCGCGCAU	22	197	11	scaffold_2:6713696:6713892	+	−98.80
LC-m0066	GGAGCAUCAUCAAGAUUCACA	-	21	105	7	scaffold_2:22285766:22285870	+	−51.40
LC-m0085	-	TCTGGTAGATAGAGGCTTGCAGA	23	343	15	scaffold_31:20483:20825	+	−78.41
LC-m0116	-	UCUUAGUUGGCAUCAGGGGAG	21	89	18	scaffold_3:15171393:15171481	−	−47.20
LC-m0129	-	GAUUCGAUGGAUCGUGAAUCGGA	23	91	9	scaffold_4:18011756:18011846	+	−25.90
LC-m0207	CAGTTTGGTTCGGTTCGGTTTTA	-	23	273	8	scaffold_7:9303778:9304050	−	−59.03
LS-m0011	GUGGGUUGGUCACGAUCUGGACA	-	23	73	7	scaffold_1:23025602:23025674	+	−22.10
LS-m0017	GGGUGAGAGGUUGCCGGAAAGA	UUUCCGAAACCUCCCAUUCCAA	22	120	233	scaffold_1:29646062:29646181	+	−47.20
LS-m0064	UAAAAGAAAAGGAUGUGCUAA	-	21	96	12	scaffold_2:22728227:22728322	+	−56.10
LS-m0068	UCAUUAUAAAGGAGCUGAGCG	-	21	81	18	scaffold_2:3509153:3509233	−	−37.20
LS-m0071	UCAUUAUAAAGGAGCUGAGCG	-	21	81	18	scaffold_2:3663645:3663725	−	−35.10
LS-m0072	UCAUUAUAAAGGAGCUGAGCG	-	21	81	18	scaffold_2:3702370:3702450	−	−35.10
LS-m0146	-	ACCCCGGGAAGCACACCAUUC	21	104	32	scaffold_4:24898628:24898731	+	−76.80
LS-m0229	AUUUUGACAAUUCCGGUGACG	UCACCGGAAUUGUCAAAAUGA	21	112	70	scaffold_7:18921383:18921494	+	−65.80
RC-m0001	UGCGUGGCGUAGGUUAUCCGG	GUGUUGAUUGUACCGGCGUAUG	22	84	9	scaffold_10:842623:842706	−	−36.60
RC-m0006	UGGGUUUAUAAGGAGUUGAGCUA	-	23	94	10	scaffold_16:367482:367575	−	−44.80
RC-m0010	-	ACGUGUCAAGUGUUGAGAAUGGU	23	132	12	scaffold_1:4213465:4213596	+	−73.00
RC-m0027	GGGUGAGAGGUUGCCGGAAAGA	UUUCCGAAACCUCCCAUUCCAA	22	120	181	scaffold_1:29646062:29646181	+	−47.20
RC-m0030	-	GAAAUUUCGUCGGGAAAGGUU	21	84	11	scaffold_1:30447571:30447654	+	−26.40
RC-m0032	UGUAAUCCAAGAGAUCAGGACUG	GUCUCGAUCUCUUAGACCACAGG	23	75	11	scaffold_1:31233982:31234056	+	−34.30
RC-m0063	GCTTCTATCTCTTCCTTTAGC	TGAAGGAAGAGATAGAAGCGC	21	127	264	scaffold_1:41019402:41019528	−	−79.00
RC-m0146	TTACATACTTCTAATCTCGGCT	-	22	116	66	scaffold_4:14813352:14813467	+	−64.90
RS-m0062	-	UAUGGCAGGAAAGAAUGUGA	20	85	31	scaffold_2:2899244:2899328	+	−42.10
RS-m0084	AAAAGTATTACACGTCGGTTACA	-	23	344	113	scaffold_2:3555213:3555556	−	−75.30
RS-m0110	CGUGGUAUCAGAGUCAUGUUA	-	21	100	9	scaffold_3:8575410:8575509	+	−42.00
RS-m0136	-	UCUUAGUUGGCAUCAGGGGAG	21	89	38	scaffold_3:15171393:15171481	−	−47.20
RS-m0154	-	ACCCCGGGAAGCACACCAUUC	21	104	147	scaffold_4:24898628:24898731	+	−76.80
RS-m0177	-	UUAUUGGUCGGGGAUAGCAAA	21	104	288	scaffold_5:190969:191072	+	−56.80
RS-m0189	GGATTGTTTAGTGTGCGGATT	TCCGCATGCTAAACAATCCGG	21	206	180	scaffold_5:316931:317136	−	−84.10
RS-m0263	AUCAUGUCACCAGGAACCAAG	UGAUUCUUGUUGACGUGAUGU	21	104	2085	scaffold_7:2887134:2887237	−	−69.40

* The location of the hairpin precursor(s) on reference of *Prunus persica* genomic scaffolds.

### Genome-wide expression patterns of drought-responsive miRNAs identified in peach

**Figure 3:**
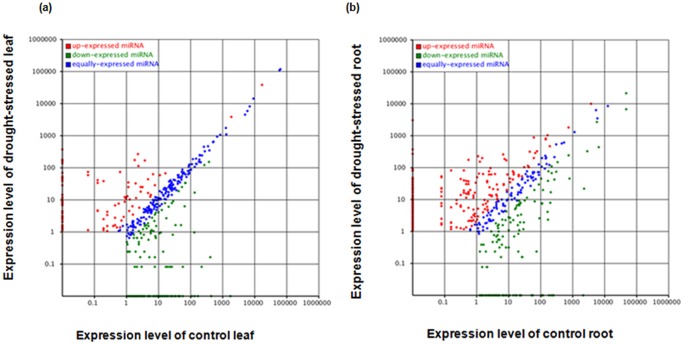
Scatter-plot graphs represent the miRNA differential expression patterns between control and drought stress in both leaves (a) and root (b). The X axis indicates normalized gene expression levels in control and the Y axis indicates the normalized gene expression levels (per transcript) in drought -stresses tissues. The dots which are located at the upper and lower side of the diagonal line reflects the changes in the expression levels of miRNA genes; above the diagonal line, indicating up-regulation whereas below the diagonal line indicating down-regulation. For miRNA deep-sequencing experiment, the fold change cut-off was set at 1.5.

**Figure 4:**
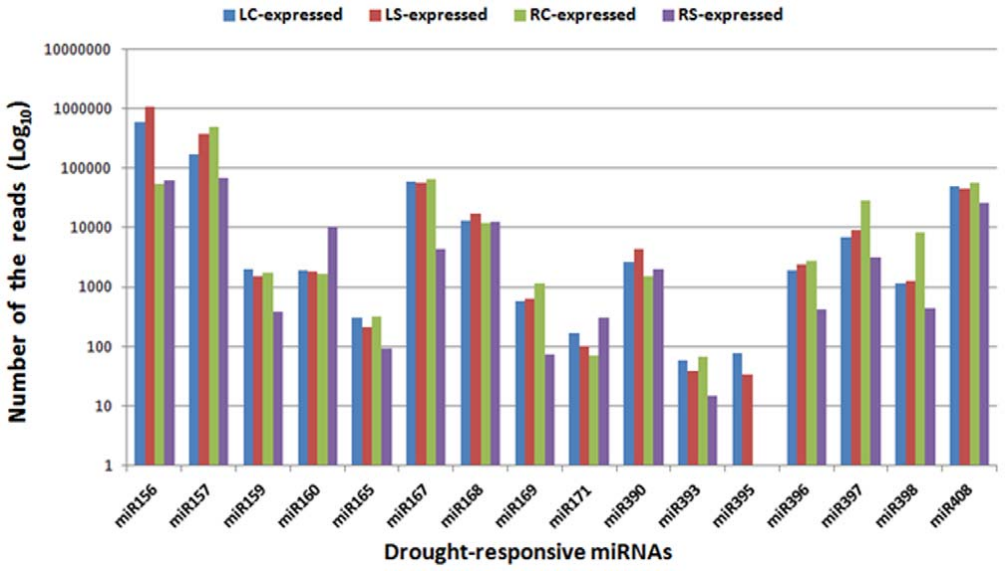
The normalized expression level of drought-responsive miRNAs in each library.

262 and 368 miRNAs were observed with more than 2 fold change response to drought treatment in peach leaf and root, respectively (Table S6; Figure 3). As reported in previous studies [9], [42], [53], [66]–[72], a series of miRNAs, including miR156, miR159, miR160, miR165/miR166, miR167, miR168, miR169, miR170/miR171, miR390, miR393, miR395, miR396, miR397, miR398, and miR408 are considered to be drought-responsive miRNAs (Figure 4). Our analysis also revealed that miR165 and miR167 were commonly down-regulated in both leaf and root, whereas miR156 was slightly up-regulated in root and leaf after stress treatment. The expression level of miR159, miR169, miR393, miR397, miR398 and miR393 were only decreased in root under drought stress while the miR395 were only down-regulated in leaf in response to drought (Figure 4). The miR160 were solely up-regulated in root, whereas there were no changes in the expression level of miR168, miR390 in leaf and root tissues (Table 5). Since our results largely agree with previous studies (well reviewed in [7], [8]), it has been said that peach has its own specific miRNA expression profile under drought-stress.

**Table 5:** The expression level of drought-responsive miRNAs (also, evolutionary conserved) in both leaf and root libraries of *P. persica*.

miRNA	LC-expressed	LS-expressed	Up/Down	RC-expressed	RS-expressed	Up/Down
miR156	934112	1346985	**↓** *(slightly up-regulated)*	68360	81033	**↓** *(slightly up-regulated)*
miR157	262981	465762	**↑** (Up-regulated)	606233	87444	↓*(down-regulated)*
miR159	3033	1889	Not significantly changed	2176	489	↓*(down-regulated)*
miR160	2969	2235	Not significantly changed	2118	13332	↑(up-regulated)
miR165	476	261	**↓** *(down-regulated)*	405	119	↓*(down-regulated)*
miR167	92812	71576	↓*(down-regulated)*	83715	5571	↓*(down-regulated)*
miR168	20085	21168	Not significantly changed	14751	16549	Not significantly changed
miR169	907	804	Not significantly changed	1420	94	↓*(down-regulated)*
miR171	262	128	Not significantly changed	90	393	↑(up-regulated)
miR390	4098	5471	Not significantly changed	1862	2659	Not significantly changed
miR393	91	48	Not significantly changed	83	20	↓*(down-regulated)*
miR395	120	42	**↓** *(down-regulated)*	Not detected in root library		
miR396	2993	3033	Not significantly changed	3388	539	↓*(down-regulated)*
miR397	10464	11428	Not significantly changed	36291	4151	↓*(down-regulated)*
miR398	1741	1569	Not significantly changed	10259	586	↓*(down-regulated)*
miR408	77621	55268	Not significantly changed	71947	34715	↓*(down-regulated)*

As seen in the table, most of the drought-responsive miRNAs were markedly down-regulated except of miR156. Comprehensive information about the fold-change (log2), p-value, expression level of libraries with normalized value can be found in Table S6.

### Target prediction and function analysis

**Figure 5:**
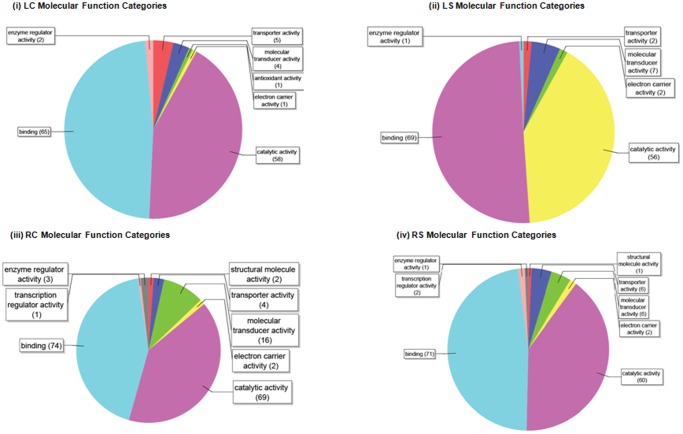
miRNA target transcripts molecular function and biological process categories. (**A**) The pie diagrams demostrating the significant number of putative peach miRNA targets within the molecular function categories based on the Blast2Go data mining. As shown in Figure 3, the GO hits pertaining to binding and catalytic activity function was overwhelmingly dominant component of all hits. (**B**) Pie chart illustrating the composition of miRNA-target transcripts (GO term) of each library in the biological-processes categories.

The putative miRNA targets in peach were predicted using BlastN search (v2.2.22) against EST and cDNA sequences in *P. persica* genome annotation database (http://www.plantgdb.org/XGDB/phplib/download.php?GDB=Pe) based on the rules described in the section of methods. Based on this strategy, a total of 672, 1293, 1194 and 1719 putative miRNA targets were obatined for LC, LS, RC and RS libraries, respectively. Although the number of targets for each miRNA varied considerably among the libraries ranging from 65 to 1 for LC, from 457 to 1 for LS, from 433 to 1 for RC and from 563 to 1 for RS, most of miRNAs in each library have only one predicted target transcripts; LC (30%), LS (30%), RC (32%) and RS (37%) (Table S5). The sequence alignment between miRNA and its predicted target genes are also found in Tables S4 and S5. For comprehensive annotation, all putative target transcripts in each library were analyzed by Gene Ontology (GO) terms with the aid of Blast2GO program with default parameters. Among the peach miRNA targets identified, a total of 571 target transcripts (137 for LC, 133 for LS, 148 for RC and 153 for RS) generated significant GO terms for further analysis. Then, transcripts representing genes with a known function were categorized by biological process, cellular component and molecular function according to the ontological definitions of the GO terms. The putative target transcripts of miRNAs in the biological process category were related to binding (65 terms for LC, 69 terms for LS, 74 terms for RC and 71 terms for RS), catalytic activity (58 terms for LC, 56 terms for LS, 69 terms for RC and 60 terms for RS), electron carrier activity (1 term for LC, 2 terms for LS, RC and RS), antioxidant activity (1 term for LC), molecular transducer activity (4 terms for LC, 7 terms for LS, 16 terms for RC and 6 terms for RS), transporter activity (5 terms for LC, 2 terms for LS, 4 for RC and 6 terms for RS), structural molecule activity (2 terms for RC and 1 term for RS), enzyme regulatory activity (2 terms for LC, 1 term for LS and RS and 3 terms for RC) and transcription regulator activity (1 term for RC and 2 terms for RS) (Figure 5A). As shown in Figure 5, most of the miRNA target genes were assigned to the binding category whose present sequences appear to be involved in nucleic acid binding, protein binding and ion binding. Because these sequences encode transcription factors, this is in accord with previously reported notion explained as a large proportion of miRNA targets encode transcription factors [7], [8], [14]. In the biological-processes category, total number of miRNA target sequences in each library fell into multiple classes, however, it is notable that some of these transcripts in the biological process category were related to stress response process, for example; response to stress (13 terms), oxidation-reduction process (9 terms) and response to abiotic stimulus (8 terms) for LC, oxidation-reduction process (12 terms), response to abiotic stimulus (7 terms), small molecule metabolic process (10 terms) and cellular catabolic process (6 terms) for LS, response to salt stress (7 terms), small molecule metabolic process (7 terms) and oxidation-reduction process (12 terms) for RC and oxidation-reduction process (6 terms), cellular response to stress (7 terms), response to salt stress (5 terms) and catabolic process (5 terms) for RS (Figure 5B).

Based on KEGG biochemical pathway analysis, a total of 351, 530, 563 and 597 target transcripts involved in different cellular pathways were determined for LC (124 pathways), LS (216 pathways), RC (211 pathways) and RS (226 pathways) libraries, respectively (Table S7). The pathway analysis for all four libraries show that target transcripts are commonly involved in some cellular pathways including: plant-pathogen interaction (18.75% for LC, 8.73% for LS, 9.95% for RC and 8.23% for RS), metabolic process (6.08% for LC, 8.40% for LS, 8.30% for RC and 7.70% for RS) biosynthesis of secondary metabolites (3.89% for LC, 4.41% for LS, 4.31% for RC and 4.19% for RS) and plant hormone signal transduction (4.22% for LC, 2.99% for LS, 3.60% for RC and 3.29% for RS). It is interesting to note that most of target genes were associated with plant hormone signal transduction because the environmental stress factors such as drought affect plant hormone balance and these biotic environmental stress factors are regulated by transcription factors, which are potential targets of most plant miRNAs.

### qRT-PCR validation of *P. persica* miRNAs and target transcripts

**Figure 6:**
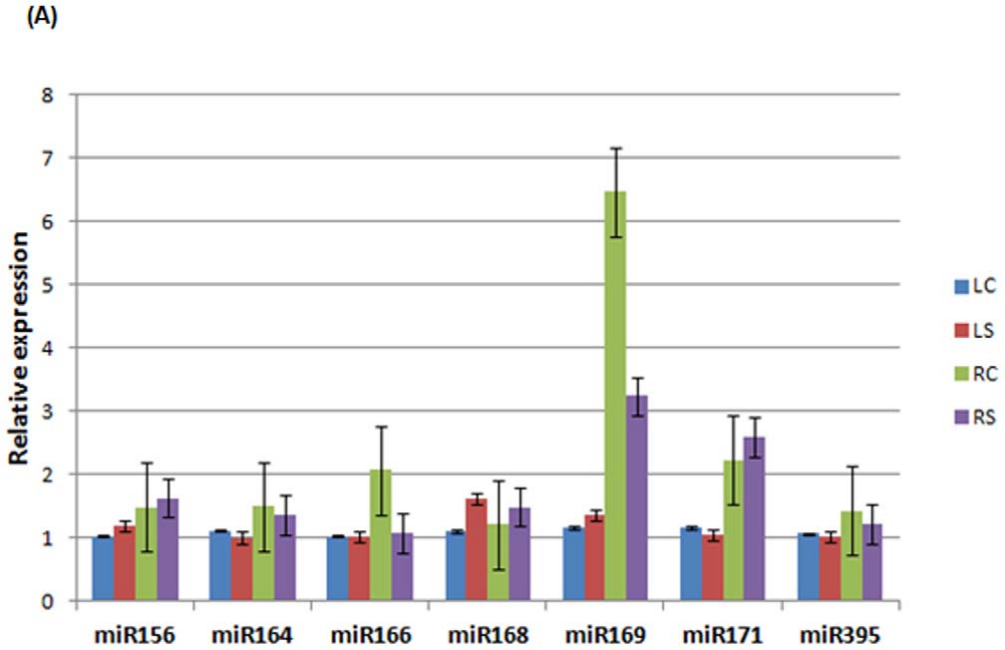
qRT-PCR validation of selected drought-responsive *P. persica* miRNAs and their target genes. (**A**) Relative expression level of drought-responsive miRNAs as determined by stem-loop RT-qPCR. (**B**) Relative quantification of target genes of six drought-responsive miRNAs by qRT-PCR.

We applied stem loop quantitative real-time RT-PCR (qRT-PCR) for further experimental verification of the presence of some conserved miRNAs and comparison of the expression pattern of these miRNAs with deep sequencing. Analysis of seven drought-responsive miRNAs by qRT-PCR show that the expression level of miR156 and miR168 were high in leaves and roots under drought stress in comparison to control samples while the expression of miR164 and miR395 was down-regulated in root and leaf tissues of drought-stressed samples. The expression of miR169 was induced in leaf but inhibited in root tissues after drought treatment, whereas the expression level of miR171 was induced in root but inhibited in root tissues under drought. As for miR166, although its expression was down-regulated in root tissues in response to drought, the expression level of miR166 were not changed between control and drought stressed leaves of peach (Figure 6). The relative expression profile of miR156, miR164, miR168, miR171 and miR395 using qRT-PCR had a good correlation with deep sequencing. However, there is a discrepancy between the results obtained from deep sequencing and qRT-PCR experiment. Deep sequencing results showed that the expression of miR169 was down-regulated in leaf tissue while qPCR experiment revealed that its expression was up-regulated in leaf tissue after treatment. Rather than experimental methods, duration of drought probably caused expression level differences.

qRT-PCR was also used for detection and quantification of predicted targets of six drougt-responsive miRNAs (miR156, miR164, miR166, miR169, miR171 and miR395). Our results revealed a negative correlation between the levels of miRNAs and those of their target messages (Figures 6A and 6B). Thus, down-regulation of miRNA could lead to increased expression of its target gene. For instance, the decreased expression of miR164 and miR395 promoted the expression of their targets genes in both root and leaf tissues. Conversely, drought-induced up-regulation of miR156 led to down-regulation of its target gene.

## Discussion

Among various abiotic stresses, drought is considered to be one of the most detrimental factors to agriculture and adversely influence crop productivity and quality due to its high scale of impact and wide distribution [73]. As land plants are sessile organisms, they cannot escape from unfavourable environmental stress conditions surrounding them. Thus, land plants have to develop various mechanisms at the physiological and molecular levels in order to cope with stress. Recently, miRNAs have turned out to be new players in plant tolerance to environmental stresses like drought, cold, heat and high salinity [74] and much effort has been devoted to understanding their role in the responses of drought stress in various plants including; *Hordeum vulgare* [9], *Medicago truncatula* [66], *Populus euphratica* [68], *Vigna unguiculata* [69] and *Oryza sativa* [75]. In the present study, deep sequencing technology was used for quantitative determination of genome-wide miRNA expression patterns of *P. persica* in response to drought. A total of 535 known miRNAs were detected in peach, although 126, 256, 293, 329, 197, 157 and 126 known miRNAs were identified in *M. truncatula* [66], *P. euphratica* [68], *V. unguiculata* [69], *A. hypogaea* [76], *G. max* [77], *P. aphrodite* [78] and *V. amurensis* [79], respectively. Hence, it can be possible to deduce that the small RNA repertoire of peach is relatively richer than other plant species. By comparing the expression level of individual peach miRNAs in drought-stressed tissues to control, the expression level of 262 (104 up-regulated, 158 down-regulated) of the 453 miRNAs significantly changed in leaf tissue, whereas 368 (221 up-regulated, 147 down-regulated) of the 465 miRNAs had expression levels that significantly changed in root tissue (Table S6). Among these miRNAs, drought-responsive miRNAs (Table 5, Figure 2) were differentially expressed and showed fluctuations in their expression in both peach leaf and root. The expression of miR165 and miR167 was found to be significantly down-regulated in leaf and especially root under drought stress, whereas miR156 was slightly but not significantly up-regulated in drought-stressed tissues. It should be noted that expression of miR165/166 was induced in leaf but inhibited in root tissues of *H. vulgare* after dehydration stress [9] while miR166 was down-regulated in both leaf and root tissues of *P. persica*. Similarly, miR171 expression was up-regulated in leaves of barley while transcript level of miR171 were only increased in the root, but not in the leaf of peach in response to drought. Although miR167 was significantly down-regulated in leaf and root libraries of peach, its up-regulation was observed in the *Arabidopsis thaliana* [42] and *P. euphratica* [68]. Some miRNAs in different plant species display different expression patterns in response to drought; for example, miR168 is up-regulated and down-regulated in *A.thaliana* [42] and *O.sativa* [80], respectively while its expression level was not significantly affected by drought. As another drought-responsive miRNA, miR395 was down-regulated and its expression was restricted to root tissue and was not detected in leaves of peaces (Table 5). As the expression levels of miR159, miR396 and miR397 were down-regulated in peach after treatment, this finding is inconsistent with previous reports suggesting that expression of these miRNAs was up-regulated in both *A. thaliana* and *P. euphratica* [42], [68]. Although the measured expression level of the miR166 did not change in leaf tissue of peach under drought stress, the qPCR results indicate that its expression level decreased in root tissue after treatment (Figure 6). This result is consistent with the previous finding that the miR166 was downregulated in roots of barley after drought stress [71].

Because the function of miRNAs appears to be in gene regulation by targeting specific mRNAs for degradation or translation inhibition, identifying the potential target transcripts of miRNAs is crucially important for understanding miRNA-mediated processes such as drought tolerance in plants. Therefore, target prediction analyses were particularly conducted for drought-responsive miRNAs, mentioned above, whose targets generally encode transcription factors and tranporters. Among them, miR159 was up-regulated in response to water limitation and was confirmed to target MYB transcription factors (myb33 and myb101) in *Arabidopsis* under drought stress in response to ABA accumulation [81]. However, in contrast to *Arabidopsis*, miR159 was down-regulated in rice [80] and peach root tissue. Although some members of MYB-family transcription factors were found in peach transcriptome libraries (at GDR; Genome database for Rosaceae), we could not determine the Myb transcription factors as targets for miR159 and this result may be consistent with previous findings that miR159 target was not related to MYB in tomato [82]. Another miRNA, miR160 is known to target three Auxin Response Factors (ARF 10, ARF 16 and ARF 17) in *Arabidopsis* [83] and it was reported that ARFs are transcription factors binding to auxin-responsive promoter elements to induce or repress auxin-regulated transcription [84] during the plant development such as root development and branching. After drought treatment, miR160 was up-regulated in peach roots and this miRNA (miR160a and miR160b) was also up-regulated in drought-tolerant cowpea cultivar in response to drought [69]. Thus, the upregulation of miR160 could be important in drought responses among different plant species. It was revealed that the miR169 family members were associated with drought response and high salt stress [52], [55]. The miR169 targets a gene family encoding the alpha subunit of CCAAT-binding NFY transcription factors (NFYA) requiring for adoptation to drought stress [8]. Two members, expression level of miR169a and miR169c were substantially down-regulated in *Arabidopsis* via ABA-dependent way [52] and also showed that level of miR169 was decreased under drought stress in *M. truncatula* by using high-throughput sequencing and qRT-PCR methods [66]. In addition, expression level of miR169 increased in two cowpea genotypes [69]. These results show good concordance with our findings that miR169 was down-regulated in peach after treatment. However, miR169g was up-regulated in rice during drought stress because the miR169g promoter contains two putative DRE (dehydration-responsive) *cis*-elements, causing the upregulation in response to drought and cold [53]. As an abiotic stress, drought disturbs the balance between ROS production and ROS elimination and thus leads to ROS accumulation in plant cells, which damages nucleic acids, oxidizes proteins and causes lipid peroxidation [85]. The detoxification of ROS radicals were carried out by Superoxide dismutases (SODs). The miR398 regulates the expression level of two Cu/Zn superoxide dismutases (cytosolic CSD1 and chloroplastic CSD2) under drought stress and level of miR398 was down-regulated in both *M. trunctula* [66] and maize [70], whereas its up-regulation was found in *Triticum dicoccoides* after 8-h stress [71]. In this study, we found that the level of miR389 was down-regulated in peach after stress treatment. A recently published paper [62] showed that miR408 are up-regulated in response to water deficit in *M. truncatula* by targeting plantacyanin, and this miRNA was also induced in leaf tissue of *H. vulgare* under dehydration stress [9]. Contrary to *M. truncatula* and *H. vulgare*, expression level of miR408 decreased in peach and this result is consistent with previous finding [67] where they also detected the induction in expression of miR408 upon drought stress in *O.sativa*. However, it is necessary to specify that plantacyanin, putative target of miR408, was not found in peach transcriptome library during the computational target prediction process, therefore experimental methods such as RLM-RACE or degradome sequencing may be used for accurate determination of miR408 target.

## Conclusions

In the present study, we genome-widely identified miRNAs and their expression pattern of drought-responsive miRNAs in roots and leaves of *P. persica* by using high-throughput sequencing. The expression level of 262 (104 up-regulated, 158 down-regulated) of the 453 miRNAs for LC/LS and 368 (221 up-regulated, 147 down-regulated) of the 465 miRNAs for RC/RS changed significantly in response to drought. Among them, drought responsive miRNAs (miR156, miR164, miR166, miR168, miR169, miR171, and miR395) were detected and their expression levels were measured by qRT-PCR. Our research also represents the first concerted effort to determine the large-scale small RNA datasets of peach. After sequencing, we identified a total of 531, 471, 535 and 487 known miRNAs and a total of 197, 221, 238 and 265 novel miRNA candidates for LC, LS, RC and RS tissues, respectively. Most of putative target transcripts for these miRNAs in biological process were related to nucleic acid binding (transcription factors) and catalytic activity. These results will greatly contribute to the understanding of post transcriptional gene regulation response to drought stress in peach.

## Materials and Methods

### Plant materials and stress treatment

*P. persica* cultivar Francoise plants obtained from *in vitro* culture clones were grown in plastic pots for one month [86]. Drought-stress treatments were applied to the plants with similar stem lenght and leaf area for one week by withholding the water until leaves-wrinkle in greenhouse conditions as +24/18°C day/night 16/8 h light/dark. Then, root and leaf tissue samples were collected and immediately frozen in liquid nitrogen and stored at −80°C until RNA isolation.

### Total RNA Isolation, small RNA library construction and sequencing

Total RNAs from leaves and roots of control samples and plants exposed to drought stress used in this study was isolated using an TriPure Isolation Reagent (Roche) according to the manufacturer's protocol. The quality and quantity of purified RNA were assessed by using a Nanodrop ND-2000c spectrophotometer (Nanodrop Technologies, Wilmington, DE, USA). Then, DNase treatment was carried out as described before [9] and all samples were stored at −20°C for miRNA quantification. For each control and stress treatment samples, equal amount of total RNA was pooled from three biological replicates to generate enough RNA (approximately 1000 ng) for deep sequencing. *P. persica* small RNA library construction, cluster generation and deep sequencing were carried out by the BGI (Beijing Genomics Institute, Hong Kong). Briefly, the isolated total RNAs of each sample was resolved on a denaturing 15% polyacrylamide gel for size selection and these small RNAs (≤30 bases) were ligated to a pair of Solexa adaptors at the 5′ and 3′ ends using T4 RNA ligase. After ligation and purification, adapter-ligated small RNAs were reverse transcribed and 15-cycles amplified (cDNA RT-PCR) with a pair of adapter complementary primers in order to produce sequencing libraries. Then, PCR products were purified and were directly sequenced using Illumina HiSeq 2000 instrument according to manufacturer's recommendation (BGI, Shenzhen, China).

### Bioinformatics analysis of sequencing data and novel miRNA prediction

After the sequencing reactions were complete, the high-quality small RNA reads ranging from 18 to 30 nucleotides were obtained from raw data analysis pipeline including; removing the low quality tags and trimming adaptor sequences so as to identify conserved and novel miRNAs in *P. persica*. Then, small RNA reads were used as queries to search against the Rfam family database and NCBI Genbank database to remove non-coding RNAs such as rRNA, tRNA, snRNA, snoRNA; the remaining sequences were searched against the miRBase database v18.0 with up to two mismatches to idnetify “conserved” mature miRNA orthologs. small RNAs not mapped to any miRNAs in miRBase were subsequently analyzed for potenial novel miRNAs by the program MIREAP (developed by BGI) with default parameters for mapping the peach genome and obtaining all candidate precursors with hairpin-like structures of novel miRNA candidates. Secondary structures of novel miRNAs were also checked using Mfold 3.2 [87].

### Identifying miRNAs respoisve to drought treatment

In order to identify drought-responsive miRNAs and determine their genome-wide expression changes in response to drought, we first compared the gene expression patterns of miRNAs in control and the drought-treated leaf and root tissues in peach. Towards this purpose, we considered the following criteria; (i) adjusted p-value should be less than 0.01 (p-value<0.01) in at least one data set, (ii) fold change or log_2_ ratio of normalized counts between drought and control libraries was greater than 1 or less than −1 in one of the libraries. The frequency of miRNA read counts was normalized as transcripts per million (TPM) and normalization of miRNA expression levels between control and drought-stresses samples was carried out based on the following formula:
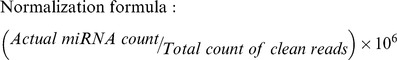
Actual read counts and normalized counts for each miRNA in each library are provided (Table S6). Afterwards, the fold-change between treatment and control and P-value were calculated from the normalized expression using the formula shown below:
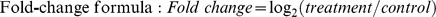


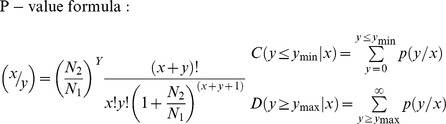
Poisson distribution model was used for estimating the statistical significance of miRNA expression changes under control and treatment conditions. Up-regulation of any miRNA expression levels was considered a positive value while negative values indicate down-regulation

### miRNA validation and quantification by quantitative stem-loop RT-PCR

For miR156, miR164, miR166, miR168, miR169, miR171 and miR395, the miRNA stem-loop reverse transcription reaction was performed in a volume of 10 µL containing 2, 20, and 200 ng of total RNA samples of leaf and root samples (1 µL), 0.5 µL 10 mM dNTP mix, 1 µL stem-loop RT primer (1 µM) and 7.5 µL nuclease free water. All those components of the reaction were mixed singly for the different dilutions of total RNA stem-loop RT primer cDNA syntheses and incubated for 5 min at 65°C and then put on ice for 2 min. Then, 4 µL first strand buffer (5×), 2 µL 0.1 M DTT, 0.1 µL RNAseOUT (40 units/µL) and 0.25 µL SuperScript III (200 U/µL; Invitrogen) were added onto each tube and RT reaction temperature program set as follows: 30 min at 16°C, 60 cycles at 30°C for 30 s, 42°C for 30 s, and 50°C for 1 s. The RT reactions were terminated at 85°C for 5 min. During cDNA synthesis for miRNA quantification, we also generated additional reaction tubes including all components without RT primer (no-RT) and RNA template (no-RNA) as control reactions.

To experimentally validate predicted *P. persica* miRNAs and to measure and compare the expression levels of the miRNAs in leaf and root tissues on different stress conditions, qRT-PCR was carried out via FastStart SYBR Green Master mix (Roche) on The LightCycler ® 480 II Real-Time PCR (Roche). By using previously synthesized 2 µL RT stem–looped cDNA products, quantitative PCR reactions were performed as followed; 10 µL 2× PCR Master mix, 1 µL forward (10 pmol), 1 µL reverse (10 pmol) primers, 0.3 µL (30 nM) reference dye and 10.7 µL nuclease-free water were mixed. With specifically designed forward primers for each individual miRNAs, the universal reverse primer (5′-GTGCAGGGTCCGAGGT-3′) [30] (Table S8) was designed for all the quantifications. Specified qRT-PCR thermal setup was adjusted as follows: 95°C for 15 min, followed by 40 cycles of 95°C for 5 s, 56°C for 10 s and 72°C for 30 s. All PCR products were denatured at 95°C and cooled to 65°C and the fluorescence signals were accumulated consistently from 65°C to 95°C as the temperature increased at 0.2°C per second. The reactions were repeated at least three times for credible statistical analysis.

### Target transcript validation

In order to validate and detect expression level of predicted miRNA target genes which are related to drought stress, qRT-PCR was performed with a number of gene-specific primers. The target transcripts of Ppe-mir156, Ppe-mir166, Ppe-mir168, Ppe-mir169, Ppe-mir171, and Ppe-mir395 were obtained using psRNATarget (user-submitted transcripts and miRNA option) and BlastN algoritms. Specific PCR primers were designed using Primer3Plus software and also, primer dimers and hairpin formations were checked with the Autodimer program (Table S8). At first, complementary DNA (cDNA) was generated from 1500 ng RNA using Superscript III First-Strand Synthesis System (reverse transcriptase, Invitrogen) according to manufacturer's instructions. In brief, the qPCR was performed in a 96-well plate instrument (LightCycler® 480 Instrument II) and in 20 µl reactions that contained 1 µl of this cDNA, 300 nM each of specific forward and reverse primer, and FastStart SYBR Green I master mix (Roche). Each sample was run in biological and technical triplicates for each gene and relative quantity of these target transcrips calculated based on the housekeeping gene *18s rRNA* (forward: GTGACGGGTGACGGAGAATT/reverse: GACACTAATGCGCCCGGTAT) as a normalizer. The qRT-PCR conditions were as follows: preheating for 10 min at 95°C before 40 cycles of 95°C for 30 s, 55°C for 1 min followed by 72°C for 10 min. To eliminate false-positive results, the melting curves of the gained real-time PCR data were analysed for each run and the data of the fluorescence signal were obtained from 55°C to 95°C as the temperature increased at 0.5°C per second.

### Target Sequence annotation, Gene Ontology (GO) classification and KEGG pathway mapping

Because the majority of plant miRNAs have perfect or near-perfect complementarity with their target sites, the computational methods for finding the putative targets of miRNAs is the preferred way for prediction of conserved and novel peach miRNAs. Therefore, putative mature miRNA sequences were used as query to search against the *Prunus persica* EST database and high quality cDNA sequence by using BlastN search (http://www.plantgdb.org/XGDB/phplib/download.php?GDB=Pe). Alignments between each miRNA and its putative miRNA target(s) should meet certain criteria as follows; (i) No more than four mismatches between miRNA and its target (G-U bases count as 0.5 mismatches), (ii) No more than two adjacent mismatches in the miRNA/target duplex, (iii) No adjacent mismatches in in positions 2–12 of the miRNA/target duplex (5′ of miRNA), (iv) No mismatches in positions 10–11 of miRNA/target duplex, (v) No more than 2.5 mismatches in positions 1–12 of the of the miRNA/target duplex (5′ of miRNA) as noted by Allen [88] and Schwab [11]. The functional annotation and categorization of identified putative miRNA targets were determinated using the Blast2Go (B2G) software suite v2.3.1 with the default parameters (http://www.blast2go.com/b2ghome) [89]. Beside, these putative miRNA target sequences were used as query against the KEGG (Kyoto Encyclopedia of Genes and Genomes) database using the KEGG automatic annotation server [90] in order to reveal their biological function in various cellular metabolic pathways. With the aid of KAAS annotation tool, an ortology number (KO) in database was assigned to the genes within KEGG Genes database based on the sequence similarity comparisons and then, the KO numbers associated with the corresponding unique KEGG gene were used for mapping one of the KEGG's reference metabolic pathways.

## Supporting Information

Figure S1 **Reads abundance of various categories of small RNAs in each libraries from** ***Prunus persica*** **.** (a) Leaf control library, (b) Drought-stressed leaf, (c) Root control library, (d) Drought-stressed root.
(DOCX)

Table S1 **Summary of data cleaning of small RNA reads produced by Illumina sequencing.**
(DOCX)

Table S2 **Summary of known (conserved and non-conserved) miRNAs in libraries.**
(XLSX)

Table S3 **The graphs representing the nucleotide bias at each position of novel mature miRNA candidates.**
(DOCX)

Table S4 **Novel Prunus miRNA sequences, locations and alignments.**
(XLSX)

Table S5 **Prunus miRNA targets.**
(XLSX)

Table S6 **Read counts and normalized counts for each miRNA in each library.**
(XLSX)

Table S7 **KEGG biochemical pathway analysis of Prunus miRNA target genes.**
(XLSX)

Table S8 **List of primers used for quantification and validation of** ***P. persica*** **miRNAs and their targets.**
(DOCX)
