# Supplementary material for: Genome-Wide Identification of miRNAs Responsive to Drought in Peach (Prunus persica) by High-Throughput Deep Sequencing
Source: PLoS One. 2012 Dec 5;7(12):e50298. doi: 10.1371/journal.pone.0050298 (PMC3515591; doi:10.1371/journal.pone.0050298)

**Supplementaryl Figure S1:** Reads abundance of various categories of small RNAs in each libraries from *Prunus persica*. (a) Leaf control library, (b) Drought-stressed leaf, (c) Root control library, (d) Drought-stressed root.

**(a)** Leaf control library **(b)** Drought-stressed leaf


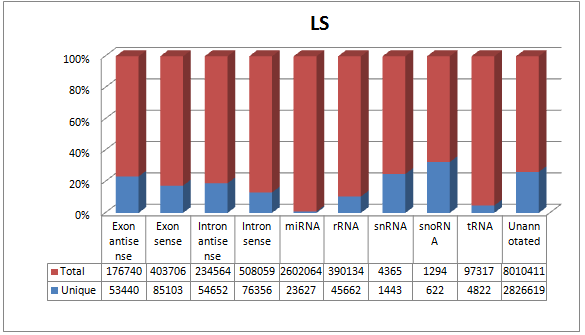

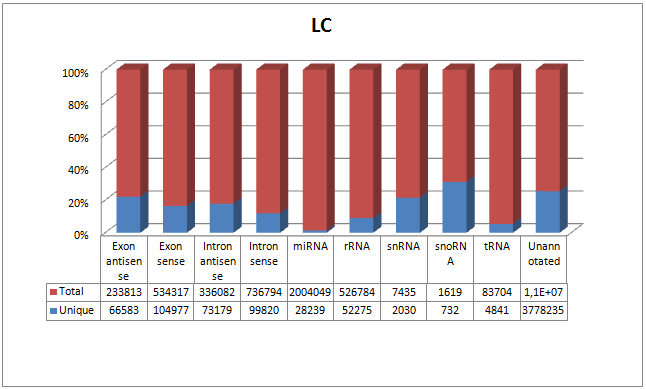


**(c)** Root control library **(d)** Drought-stressed root


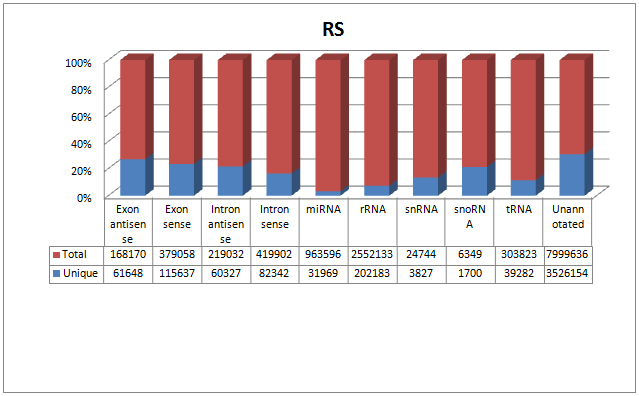

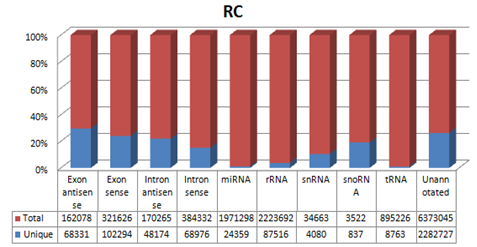

Supplement: Figure S1 — Reads abundance of various categories of small RNAs in each libraries from Prunus persica . (a) Leaf control library, (b) Drought-stressed leaf, (c) Root control library, (d) Drought-stressed root. (DOCX) [file pone.0050298.s001.docx]
