# Supplementary material for: Genome-Wide Identification of miRNAs Responsive to Drought in Peach (Prunus persica) by High-Throughput Deep Sequencing
Source: PLoS One. 2012 Dec 5;7(12):e50298. doi: 10.1371/journal.pone.0050298 (PMC3515591; doi:10.1371/journal.pone.0050298)
Supplement: Table S1 — Summary of data cleaning of small RNA reads produced by Illumina sequencing. (DOCX) [file pone.0050298.s002.docx]

**Supplementary Table S1**

Summary of data cleaning of small RNA reads produced by Illumina sequencing

| Type | | Count | Percent |
| --- | --- | --- | --- |
| Total Reads | **LC** | 15,521,503 |  |
|  | **LS** | 12,492,645 |  |
|  | **RC** | 12,726,680 |  |
|  | **RS** | 13,233,471 |  |
|  | | | |
| High Quality | **LC** | 15,499,314 | 100 % |
|  | **LS** | 12,473,137 | 100 % |
|  | **RC** | 12,703,130 | 100 % |
|  | **RS** | 13,203,304 | 100 % |
|  | | | |
| 3' Adapter Null | **LC** | 4,220 | 0.03% |
|  | **LS** | 3,473 | 0.03% |
|  | **RC** | 3,680 | 0.03% |
|  | **RS** | 3,739 | 0.03% |
|  |  |  |  |
| Insert Null | **LC** | 1,095 | 0.01% |
|  | **LS** | 1,226 | 0.01% |
|  | **RC** | 3,669 | 0.03% |
|  | **RS** | 5,427 | 0.04% |
|  | | | |
| 5’ contaminants | **LC** | 7,984 | 0.05% |
|  | **LS** | 15,137 | 0.12% |
|  | **RC** | 115,082 | 0.91% |
|  | **RS** | 80,372 | 0.61% |
|  | | | |
| Smaller than  18 nt | **LC** | 14,488 | 0.09% |
|  | **LS** | 24,503 | 0.20% |
|  | **RC** | 40,835 | 0.32% |
|  | **RS** | 76,947 | 0.58% |
|  | | | |
| Poly A | **LC** | 838 | 0.01% |
|  | **LS** | 144 | 0.00% |
|  | **RC** | 117 | 0.00% |
|  | **RS** | 376 | 0.00% |
|  | | | |
| Clean reads | **LC** | 15,470,689 | 99.82% |
|  | **LS** | 12,428,654 | 99.64% |
|  | **RC** | 12,539,747 | 98.71% |
|  | **RS** | 13,036,443 | 98.74% |
