# Supplementary material for: Genome-Wide Identification of miRNAs Responsive to Drought in Peach (Prunus persica) by High-Throughput Deep Sequencing
Source: PLoS One. 2012 Dec 5;7(12):e50298. doi: 10.1371/journal.pone.0050298 (PMC3515591; doi:10.1371/journal.pone.0050298)
Supplement: Table S3 — The graphs representing the nucleotide bias at each position of novel mature miRNA candidates. (DOCX) [file pone.0050298.s004.docx]

**Supplementary Table S3**

The graphs represented below show the nucleotide bias at each position of novel mature miRNA candidates.

**(a) Leaf Control Library (LC)**

| Position on miRNA candidate | A | U | C | G |
| --- | --- | --- | --- | --- |
| 1 | 2041 | 10528 | 4557 | 5060 |
| 2 | 6288 | 8093 | 2565 | 5240 |
| 3 | 7201 | 8205 | 3719 | 3061 |
| 4 | 5232 | 3044 | 3496 | 10414 |
| 5 | 4687 | 4151 | 4231 | 9117 |
| 6 | 7414 | 2509 | 2543 | 9720 |
| 7 | 7767 | 9726 | 1121 | 3572 |
| 8 | 7999 | 6283 | 1527 | 6377 |
| 9 | 7832 | 3855 | 4452 | 6047 |
| 10 | 8241 | 4215 | 2854 | 6876 |
| 11 | 2283 | 5729 | 5414 | 8760 |
| 12 | 4779 | 5508 | 3358 | 8541 |
| 13 | 2766 | 3237 | 5263 | 10920 |
| 14 | 3806 | 4333 | 4753 | 9294 |
| 15 | 5046 | 2929 | 5708 | 8503 |
| 16 | 8888 | 5901 | 3811 | 3586 |
| 17 | 7260 | 7941 | 2982 | 4003 |
| 18 | 2721 | 4101 | 4802 | 10562 |
| 19 | 10280 | 1922 | 5401 | 4583 |
| 20 | 4428 | 6396 | 5444 | 5918 |
| 21 | 6125 | 2531 | 2777 | 9718 |
| 22 | 5944 | 1057 | 919 | 194 |
| 23 | 1670 | 336 | 116 | 112 |


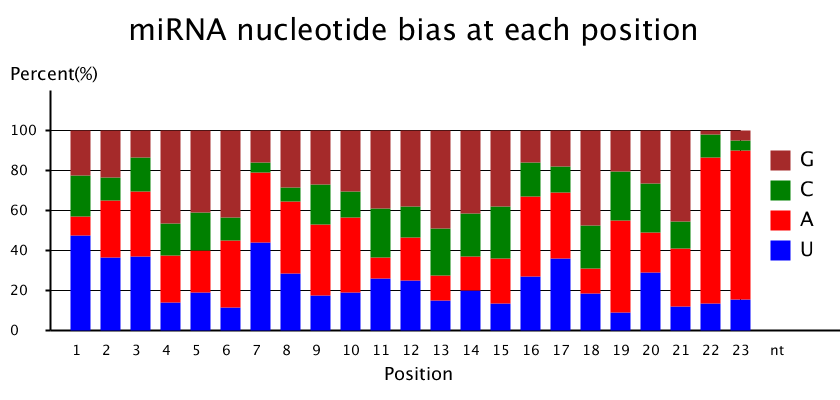


**(b) Leaf Stress Library (LS)**

| Position on miRNA candidate | A | U | C | G |
| --- | --- | --- | --- | --- |
| 1 | 5827 | 12834 | 4904 | 6764 |
| 2 | 5885 | 9489 | 3144 | 11811 |
| 3 | 13150 | 7873 | 2738 | 6568 |
| 4 | 8412 | 6508 | 5281 | 10128 |
| 5 | 7034 | 10480 | 2734 | 10081 |
| 6 | 5895 | 3304 | 6887 | 14243 |
| 7 | 7022 | 12909 | 426 | 9972 |
| 8 | 9781 | 9972 | 2939 | 7637 |
| 9 | 7728 | 3807 | 7143 | 11651 |
| 10 | 11518 | 10011 | 1257 | 7543 |
| 11 | 2055 | 12084 | 3531 | 12659 |
| 12 | 5277 | 7303 | 6300 | 11449 |
| 13 | 7214 | 5164 | 3175 | 14776 |
| 14 | 4208 | 6759 | 7099 | 12263 |
| 15 | 7756 | 3866 | 4303 | 14404 |
| 16 | 7973 | 10563 | 3569 | 8224 |
| 17 | 10322 | 6020 | 4540 | 9447 |
| 18 | 7457 | 3475 | 4350 | 15047 |
| 19 | 10621 | 5893 | 8083 | 5732 |
| 20 | 6923 | 8094 | 4410 | 10902 |
| 21 | 6461 | 5103 | 2737 | 14443 |
| 22 | 7031 | 1127 | 1456 | 282 |
| 23 | 2511 | 187 | 47 | 135 |


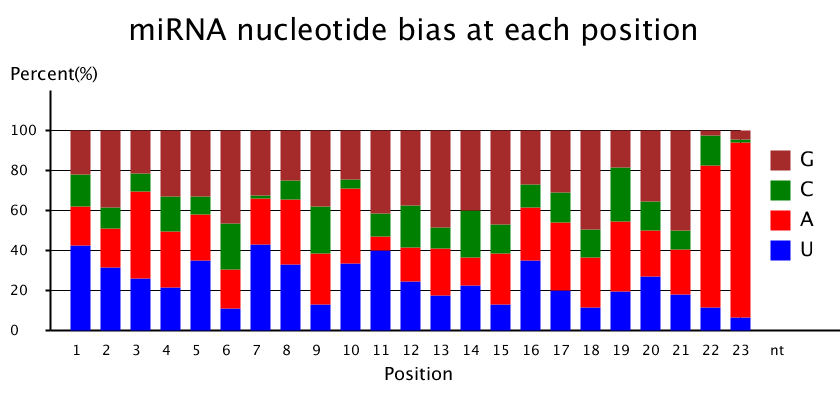


**(c) Root Control Library (RC)**

| Position on miRNA candidate | A | U | C | G |
| --- | --- | --- | --- | --- |
| 1 | 1881 | 21571 | 6265 | 4297 |
| 2 | 7583 | 6148 | 3496 | 16787 |
| 3 | 15100 | 10979 | 4882 | 3053 |
| 4 | 4732 | 12511 | 5315 | 11456 |
| 5 | 2858 | 15779 | 4650 | 10727 |
| 6 | 5941 | 3245 | 15469 | 9359 |
| 7 | 9142 | 19932 | 1235 | 3705 |
| 8 | 9213 | 15528 | 2274 | 6999 |
| 9 | 9497 | 4593 | 3653 | 16271 |
| 10 | 7455 | 13394 | 4104 | 9061 |
| 11 | 2742 | 17057 | 6984 | 7231 |
| 12 | 2189 | 7766 | 3168 | 20891 |
| 13 | 12870 | 5488 | 4706 | 10950 |
| 14 | 3366 | 4051 | 15612 | 10985 |
| 15 | 3375 | 4104 | 6213 | 20322 |
| 16 | 10571 | 16182 | 2173 | 5088 |
| 17 | 7751 | 10630 | 2711 | 12922 |
| 18 | 11764 | 3813 | 4110 | 14327 |
| 19 | 9763 | 11018 | 9218 | 4015 |
| 20 | 3150 | 9697 | 6855 | 14312 |
| 21 | 8673 | 11041 | 2968 | 10775 |
| 22 | 3212 | 1192 | 453 | 148 |
| 23 | 1279 | 136 | 83 | 222 |

**
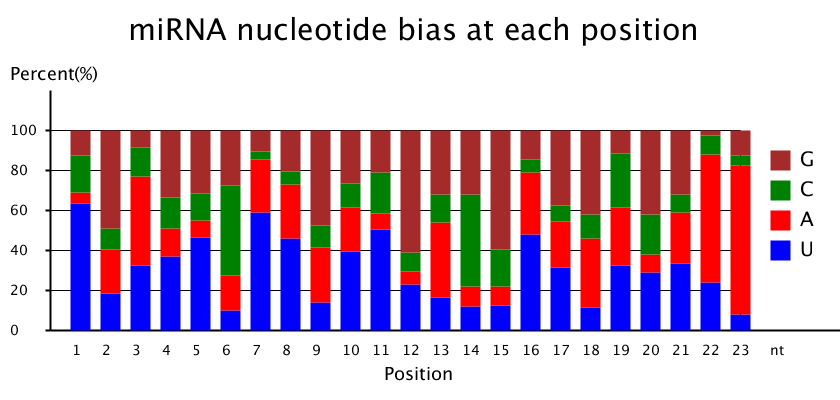
**

**(c) Root Stress Library (RS)**

| Position on miRNA candidate | A | U | C | G |
| --- | --- | --- | --- | --- |
| 1 | 3226 | 13014 | 14266 | 8280 |
| 2 | 14034 | 7464 | 3465 | 13823 |
| 3 | 13297 | 14427 | 6620 | 4442 |
| 4 | 10416 | 6339 | 5535 | 16496 |
| 5 | 3270 | 11283 | 6277 | 17956 |
| 6 | 6736 | 4088 | 5388 | 22574 |
| 7 | 8932 | 23993 | 1736 | 4125 |
| 8 | 15585 | 10960 | 2899 | 9342 |
| 9 | 17950 | 3649 | 6926 | 10261 |
| 10 | 6088 | 9757 | 6723 | 16218 |
| 11 | 3403 | 19450 | 9767 | 6166 |
| 12 | 3197 | 8541 | 2053 | 24995 |
| 13 | 6748 | 5127 | 5922 | 20989 |
| 14 | 3235 | 4929 | 8980 | 21642 |
| 15 | 4289 | 2300 | 11251 | 20946 |
| 16 | 19514 | 7863 | 4244 | 7165 |
| 17 | 17001 | 9897 | 6191 | 5697 |
| 18 | 5037 | 7093 | 2711 | 23945 |
| 19 | 20568 | 4113 | 9980 | 4125 |
| 20 | 4122 | 16070 | 7354 | 11240 |
| 21 | 8247 | 5385 | 2960 | 21697 |
| 22 | 18350 | 3387 | 1507 | 240 |
| 23 | 3996 | 210 | 81 | 697 |

**
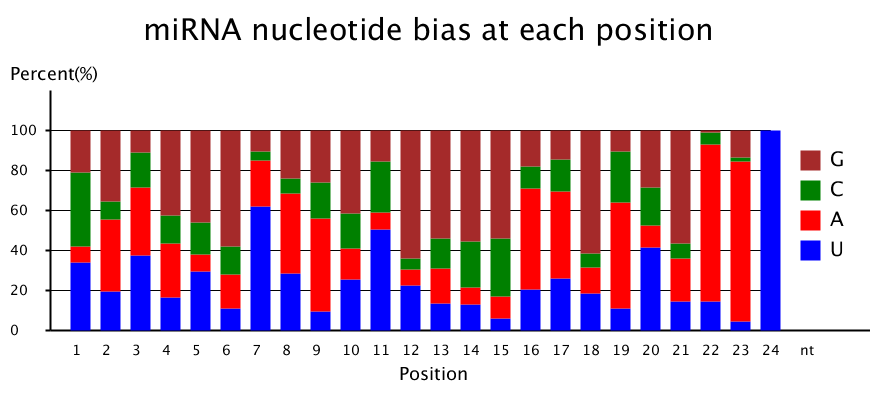
**
