# Supplementary material for: Genome-Wide Identification of miRNAs Responsive to Drought in Peach (Prunus persica) by High-Throughput Deep Sequencing
Source: PLoS One. 2012 Dec 5;7(12):e50298. doi: 10.1371/journal.pone.0050298 (PMC3515591; doi:10.1371/journal.pone.0050298)
Supplement: Table S8 — List of primers used for quantification and validation of P. persica miRNAs and their targets. (DOCX) [file pone.0050298.s009.docx]

**Supplementary Table S8**

List of primers used for quantification and validation of *P. persica* miRNAs and their targets

| miRNA sequences and miRNA quantification Primers | |
| --- | --- |
| Ppe-mir 169  Ppe-miR 169 RT  Ppe-miR 169 F | 5'-UAGCCAAGGAUGACUUGCCUGC-3'  5'-GTCGTATCCAGTGCAGGGTCCGAGGTATTCGCACTGGATACGACGCAGGC -3'  5'-GCGGCGGTAGCCAAGGATGACTT -3' |
| Ppe-miR 171  Ppe-miR 171 RT  Ppe-miR 171 F | 5'-AUUGAGCCGUGCCAAUAUC-3'  5'-GTCGTATCCAGTGCAGGGTCCGAGGTATTCGCACTGGATACGACGATATT-3'  5'-TTCCTTATTGAGCCGTGCC-3' |
| Ppe-miR 156  Ppe-miR 156 RT  Ppe-miR 156 F | 5'-UGACAGAAGAGAGUGAGCACA-3'  5'-GTCGTATCCAGTGCAGGGTCCGAGGTATTCGCACTGGATACGACTGTGCT -3'  5'-TTCCTTTGACAGAAGAGAGTG-3' |
| Ppe-miR 395  Ppe-miR 395 RT  Ppe-miR 395 F | 5'-CUGAAGUGUUUGGGGGAACUCC-3'  5'-GTCGTATCCAGTGCAGGGTCCGAGGTATTCGCACTGGATACGACGGAGTT-3'  5'-CGGCGGCTGAAGTGTTTGGGGG-3' |
| Ppe-miR 166  Ppe-miR 166 RT  Ppe-miR 166 F | 5'-UCGGACCAGGCUUCAUUCCCCC -3'  5'-GTCGTATCCAGTGCAGGGTCCGAGGTATTCGCACTGGATACGACGGGGGA-3'  5'-GCGGCGGTCGGACCAGGCTTCAT-3' |
| Ppe-miR 168  Ppe-miR 168 RT  Ppe-miR 168 F | 5’-UCGCUUGGUGCAGGUCGGGAA -3'  5'- GTCGTATCCAGTGCAGGGTCCGAGGTATTCGCACTGGATACGACATTCAC -3'  5'- TTCCTTTTCGCTTGGTGCAGGT -3' |
| Universal Reverse Primer | 5'- GTGCAGGGTCCGAGGT-3' |

NOTE: For effective amplification of these miRNAs, these forward adaptors (GCGGCGG, TTCCTT, CGGCGG) were ligated for increasing the melting-temperature (Tm).

**miRNA Target Quantification Primers**

| miRNA | Primer Sequences | Target Proteins |
| --- | --- | --- |
| Ppe-mir169  Ppe-mir169 Tar-F  Ppe-mir169 Tar-R | 5' ATCTCAGACAAACCCAACAATGTA 3'  5' CCAATCCAATCACTGACTTAACAC 3' |  |
| Ppe-mir171  Ppe-mir171 Tar-F  Ppe-mir171 Tar-R | 5' TTCCGGATCTCTTCATCAAC 3'  5' ACCATTGTCCTCCATATCCA 3' |  |
| Ppe-mir159  Ppe-mir159 Tar-F  Ppe-mir159 Tar-R | 5' GTTTAAAAGGAAACGGTTGTGTCT 3  5' GATTTTCTTCCCATTCTGTCTCAC 3' |  |
| Ppe-mir395  Ppe-mir395 Tar-F  Ppe-mir395 Tar-R | 5' CAAAACGTTCAATACCCATTTCTC 3'  - |  |
| Ppe-mir166  Ppe-mir166 Tar-F  Ppe-mir166 Tar-R | 5' CATTGCAGAAGAAACTTTAGCAGA 3'  5' CATTGTCATCTTTTGAGCAAGAAC 3' |  |
| Ppe-mir168  Ppe-mir168 Tar-F  Ppe-mir168 Tar-R | 5' CGCCCGATAGATCGAGAAA 3'  5' AGCCTCAACAGAAGCCAGAG 3' |  |
